# Piezoresistive Carbon Nanofiber-Based Cilia-Inspired Flow Sensor

**DOI:** 10.3390/nano10020211

**Published:** 2020-01-26

**Authors:** Debarun Sengupta, Duco Trap, Ajay Giri Prakash Kottapalli

**Affiliations:** 1 Department of Advanced Production Engineering, Engineering and Technology Institute Groningen, Faculty of Science and Engineering, University of Groningen, Nijenborgh 4, 9747AG Groningen, The Netherlands; debarun.sengupta@rug.nl (D.S.); d.trap@student.rug.nl (D.T.); 2 MIT Sea Grant College Program, Massachusetts Institute of Technology, 77 Massachusetts Avenue, Cambridge, MA 02139, USA

**Keywords:** cilia, biomimetic sensor, flexible electronics, carbon nanofiber, piezoresistive, flow sensor

## Abstract

Evolving over millions of years, hair-like natural flow sensors called cilia, which are found in fish, crickets, spiders, and inner ear cochlea, have achieved high resolution and sensitivity in flow sensing. In the pursuit of achieving such exceptional flow sensing performance in artificial sensors, researchers in the past have attempted to mimic the material, morphological, and functional properties of biological cilia sensors, to develop MEMS-based artificial cilia flow sensors. However, the fabrication of bio-inspired artificial cilia sensors involves complex and cumbersome micromachining techniques that lay constraints on the choice of materials, and prolongs the time taken to research, design, and fabricate new and novel designs, subsequently increasing the time-to-market. In this work, we establish a novel process flow for fabricating inexpensive, yet highly sensitive, cilia-inspired flow sensors. The artificial cilia flow sensor presented here, features a cilia-inspired high-aspect-ratio titanium pillar on an electrospun carbon nanofiber (CNF) sensing membrane. Tip displacement response calibration experiments conducted on the artificial cilia flow sensor demonstrated a lower detection threshold of 50 µm. Furthermore, flow calibration experiments conducted on the sensor revealed a steady-state airflow sensitivity of 6.16 mV/(m s^−1^) and an oscillatory flow sensitivity of 26 mV/(m s^−1^), with a lower detection threshold limit of 12.1 mm/s in the case of oscillatory flows. The flow sensing calibration experiments establish the feasibility of the proposed method for developing inexpensive, yet sensitive, flow sensors; which will be useful for applications involving precise flow monitoring in microfluidic devices, precise air/oxygen intake monitoring for hypoxic patients, and other biomedical devices tailored for intravenous drip/urine flow monitoring. In addition, this work also establishes the applicability of CNFs as novel sensing elements in MEMS devices and flexible sensors.

## 1. Introduction

Flow sensing is of utmost importance in fields related to medicine, diagnostics, pharmaceuticals, process control, weather/environmental monitoring, and forecasting systems. In the past (and, to some extent, even today), large scale mechanical sensors based on the Venturi effect, hot wire anemometry, pitot tube, and rotating turbines were employed for tasks involving flow monitoring and control. The rapid growth of the semiconductor industry and microfabrication technologies in the late 1980s led to the miniaturization of sensors in general. In 1974, Van Putten and Middelhoek [1] proposed an integrated silicon thermal flow sensor for the first time. Later, in 1985, Tai et al. [2] utilized newly emerging micromachining techniques to develop the first microscale flow sensor, which paved the way for the research and development of a new class of miniaturized micro-machined flow sensors.

Recently, researchers across various disciplines have started taking inspiration from nature to solve complex engineering and design problems. In particular, bio-inspiration has played a vital role in the development of some of the most accurate and sensitive flow sensors, inspired by cilia commonly found in many aquatic creatures, including fish. Cilia are a group of highly-sensitive hair-like natural flow sensors found in fish, crickets, spiders, and other creatures. Fluid-structure interactions that occur between the external flow and the high aspect-ratio cilia cause drag-induced bending of the cilia, which leads to the generation of electrical impulses across the hair cell membranes at their base. The biological cilia sensors in fish are known for their superior sensitivity, as demonstrated by their excellent steady-state flow velocity detection threshold, which can be as low as 10 mm/s [3].

Over the past few years, researchers have focused on developing highly sensitive MEMS/NEMS sensors inspired by biological cilia [4,5,6,7,8,9]. In most of the cases, the sensors comprised of high-aspect-ratio polymeric pillars mimicking cilia, with piezoresistive or piezoelectric sensing membranes at the bases. Some other artificial cilia flow sensors, involving hot wire anemometry and optical sensing, have also been explored by various researchers [10,11]. Inspired by biological hair cells, Chen et al. [5] developed an artificial hair cell flow sensor comprising of a silicon cantilever beam with a 700 µm tall, high-aspect-ratio SU-8 pillar at its distal tip. Experimental flow characterization of their cilia flow sensor, using a vibrating sphere (dipole) stimulus in water, demonstrated a threshold limit of 1 mm/s. In another work, Zhang et al. [12] developed a silicon-on-insulator (SOI)-based self-bent piezoresistive microcantilever flow sensor, capable of detecting flow velocities in the range of 0–20 cm/s, with a sensitivity of 1.5–3.5 Ω/ (cm s^−1^). Asadnia et al. [7] reported a MEMS self-powered flow sensor comprising of a stereolithographically fabricated polymer cilium with a Lead Zirconate Titanate (PZT) micro-diaphragm sensing element. The sensor demonstrated a high sensitivity of 22 mV/(mm s^−1^) and a very low detection threshold limit of 8.2 µm s^−1^ for oscillatory flows generated using a dipole vibrating at 35 Hz.

Most of the works reported in the past involved complex and cumbersome microfabrication techniques involving photolithography, deposition, etching, and so forth. The devices were also limited by the choice of materials that could be used in the fabrication of the high-aspect-ratio pillars to mimic the cilia and the sensing element beneath; thus, severely limiting their feasibility in flexible device applications. To address the complex fabrication issues involved with the design and fabrication of artificial cilia flow sensors, Kottapalli et al. [8,13] proposed a biomimetic hydrogel-carbon nanotube (CNT) artificial cilia flow sensor, mimicking the neuromast found in the lateral line systems of fish. The flow sensor featured a vertically aligned carbon nanotube bundle infused with hyaluronic acid (HA) hydrogel placed on a suspended piezoelectric polyvinylidene fluoride (PVDF) membrane. Oscillatory flow calibration experiments conducted on the sensor demonstrated sensitivity figures of 17.2 mV/(m s^−1^) and 45.2 mV/(m s^−1^), in air and water, respectively. Furthermore, the sensor demonstrated a detection threshold of 5 mm s^−1^ (in both air and water). Recently, Kamat et al. [9] proposed a novel workflow for fabricating complex biomimetic sensors, which entailed selective laser melting (SLM), 3D printing of an appropriate metallic mold, drop-casting polydimethylsiloxane (PDMS), and subsequent etching to release the cured PDMS structure. The sensor featured a cilia-inspired cylindrical pillar at the distal end of a cantilever, which housed a flexible microchannel with graphene nanoflakes, forming a flexible strain gauge. The sensor demonstrated a sensitivity of 30 mV/(m s^−1^) in the oscillatory flow velocity range of 65–90 mm s^−1^, with a lower detection threshold of 58 mm s^−1^. 

The key to having a highly sensitive artificial cilia flow sensor, primarily lies in the sensitivity of the sensing element and, to some extent, on the rigidity of the pillar mimicking the cilia. Recently, flexible sensors utilizing the piezoresistive properties of nanomaterial–polymer composites have gained traction in wearable device applications. Various types of nanomaterials, such as silver nanowire (AgNWs) [14], CNTs [15], carbon blacks (CBs) [16], and graphene [17,18], have been used in conjunction with elastomeric materials like PDMS, eco flex, polyimide (PI), rubber, polyurethane (PU), and so forth, to develop ultrasensitive, lightweight, and flexible sensors. In the past, our group reported electrospun carbon nanofiber (CNF) bundle-based piezoresistive sensing elements for flexible sensor applications [19]. The relative ease of fabrication of piezoresistive CNF bundles renders them an attractive choice for the development of ultrasensitive and low-powered artificial cilia flow sensors.

In this work, we report a facile method of developing an artificial cilia flow sensor, featuring a high-aspect-ratio titanium pillar mimicking the cilia, which is attached at the base to a circular CNF membrane that acts as the sensing element. The electrospun CNF bundle, sandwiched between two optically clear adhesive (OCA) films, acts as the piezoresistive sensing element. In the presence of external flow, drag-induced bending of the titanium pillar causes a displacement of the CNF membrane which, in turn, leads to a change in resistance across the membrane. The change in the resistance can be subsequently read with an appropriately designed Wheatstone bridge circuit. Steady-state air flow calibration experiments conducted on the artificial cilia flow sensor, revealed a linear sensor response with a sensitivity value of 6.16 mV/(m s^−1^). Furthermore, oscillatory flow calibration experiments conducted on the flow sensor revealed a linear response in the flow velocity range of 12-66 mm/s with a sensitivity of 26 mV/(m s^−1^) and a lower detection threshold limit of 12.1 mm/s. Also, CNF sensing elements were embedded in PDMS to develop flexible piezoresistive sensors and demonstrate the feasibility of the proposed method in wearable device applications. The facile fabrication method reported in this work will circumvent the complex fabrication methods and time budget associated with similar MEMS-based devices, which will subsequently allow for the development and commercialization of a future class of similarly inexpensive, yet accurate and sensitive, sensors.

## 2. Materials and Methods 

### 2.1. Fabrication and Synthesis of CNF Bundle Sensing Element

#### 2.1.1. Electrospinning of Polyacrylonitrile Nanofiber Bundles

The CNF bundle sensing membrane was synthesized by pyrolization of electrospun polyacrylonitrile (PAN) nanofibers. For electrospinning polyacrylonitrile (PAN) nanofibers, a 9% (w/v) homogenous polymer solution was obtained by dissolving 1.8 g of PAN powder (MW 150,000 g/mol) in 20 mL N, N-dimethylformamide (DMF) solvent. An Inovenso^TM^ NanoSpinner NE300 electrospinning setup fitted with a rotating mandrel collector (10 cm diameter) was employed for electrospinning bundled PAN nanofiber films. A standard syringe pump fixed with a 10 mL syringe was used to feed the PAN polymer solution through a needle (18 G) at a constant flow rate of 1 mL/hr. The rotating mandrel collector was covered with aluminum (Al) foil and set to rotate at a constant speed of 1500 rpm. A direct current (DC) voltage of 12 kV was applied between the collector and the conducting needle tip. A gap of 15 cm was maintained between the needle tip and base of the rotating mandrel collector. The electrospinning process was carried out for 30 min to obtain a film of uniform nanofibers deposited on the aluminum foil substrate. A constant temperature of 22 °C was maintained throughout the electrospinning process. 

#### 2.1.2. Synthesis of Electrospun Carbon Nanofibers

**Figure 1:**
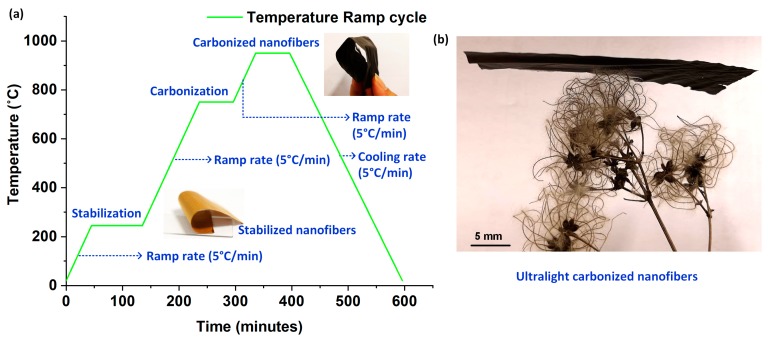
Pyrolization of electrospun PAN nanofibers to synthesize CNF bundles: (**a**) Graphical representation of the process steps involved in the synthesis of CNF bundles from electrospun PAN nanofibers; (**b**) A CNF bundle placed on a leaf to demonstrate its ultralightweight nature.

The process steps and the critical parameters involved in the stabilization and carbonization of electrospun PAN nanofibers to synthesize CNF bundles are graphically depicted in Figure 1a. The electrospun PAN nanofibers were peeled off from the aluminum foil substrate and transferred to a ceramic crucible for stabilization and subsequent pyrolization. For the first step involving stabilization, the nanofibers were heat-treated at a temperature of 245 °C for 60 min in the presence of ambient air. A constant ramp rate of 5 °C/min was used to ramp-up to the stabilization temperature. Following stabilization, in order to conduct pyrolization, the tube furnace was sealed air-tight, with a constant nitrogen (N_2_) purge for 30 min to drive out the air inside. Thereafter, the nanofibers were ramped up at a rate of 5 °C/min in N_2_ ambience to 750 °C and held for 60 min; followed by a final ramp-up at the previously mentioned rate to 950 °C, where it was plateaued for another 60 min; before ramping down to the room temperature at the same ramp rate. Figure 1b shows the photograph of a CNF bundle placed on a dried-up flower, demonstrating its ultralightweight nature. 

#### 2.1.3. Morphological Characterization of CNF Bundles

A FEI Nova NanoSEM 230 field-emission scanning electron microscope (FE-SEM) was employed to study the morphological properties of both the as-spun PAN nanofibers and synthesized CNF bundles. Gold-coated nanofiber samples with the dimensions of 2 cm × 2 cm were placed on an appropriate SEM stub, and SEM imaging studies were conducted to observe the morphological changes associated with the carbonization process. 

**Figure 2:**
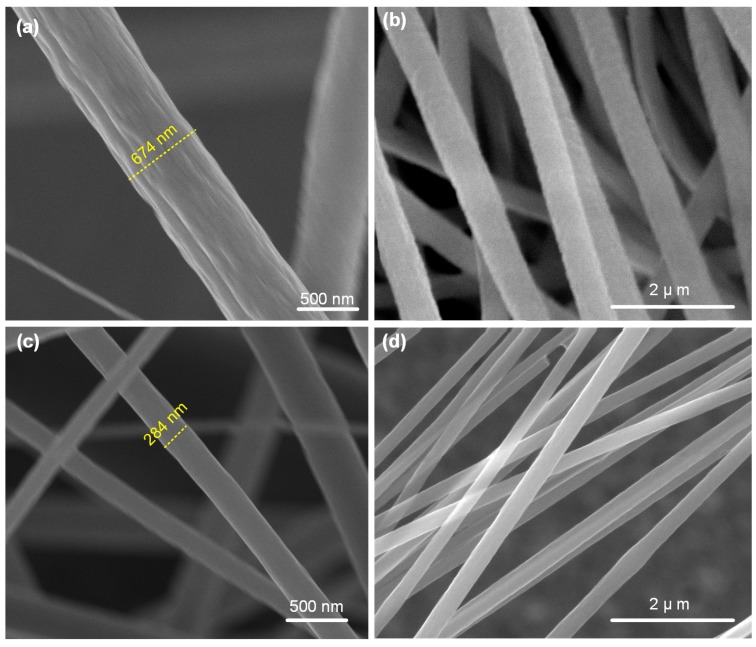
SEM micrographs of the electrospun nanofibers before and after carbonization: (**a**,**b**) Micrographs showing the as-spun PAN nanofibers at two different magnifications. The as-spun nanofibers were found to be uniform in diameter; (**c**,**d**) Micrographs showing the carbonized nanofibers with significantly reduced diameters.

The micrographs in Figure 2a,b show the as-spun PAN nanofibers. As observed from the micrographs, the electrospinning resulted in uniform nanofibers with an average diameter of 610 nm (averaged over six measurements). As evident from the micrographs in Figure 2c,d, the nanofibers showed a significant reduction in their diameter after the process of pyrolization, leading to the formation of CNFs. The CNFs were found to have an average diameter of 285 nm, which is roughly 50% of the as-spun nanofibers. The observed shrinkage in the nanofibers might arise from the breaking of carbon–nitrogen and carbon–oxygen bonds, and subsequent formation of carbon–carbon bonds, leading to the observed densification. The observed carbonization induced diameter reduction is in agreement with prior works reported in literature [20,21].

### 2.2. Cilia Flow Sensor 

#### 2.2.1. Sensor Fabrication and Sensing Principle 

**Figure 3:**
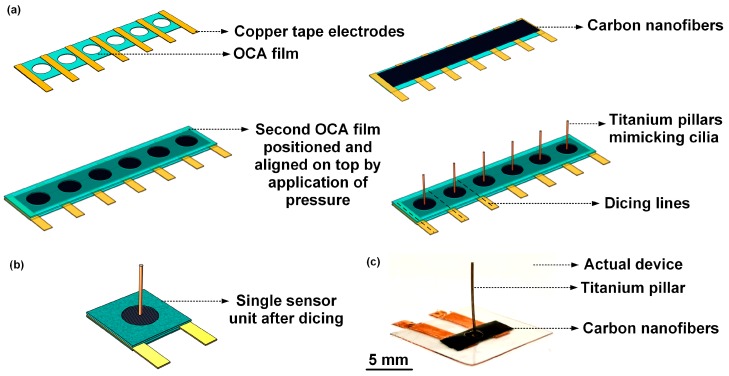
Fabrication of the cilia-inspired flow sensor: (**a**) Schematic representation of the process steps involved in the fabrication of the flow sensor; (**b**) Schematic representation of a single sensor unit after dicing; (**c**) Photograph of the actual fabricated cilia-inspired flow sensor.

A flow sensor comprising of a freestanding CNF bundle as the sensing element, and a high-aspect-ratio titanium pillar mimicking cilia, was fabricated. A circular CNF sensing membrane of 30 µm thickness and 2 mm diameter was defined through two perfectly aligned cavities formed on two sheets of OCA films, between which the CNF bundle was sandwiched. Figure 3a schematically represents the process steps involved in the fabrication of the flow sensor. Figure 3b schematically represents a single sensor unit after dicing. The photograph in Figure 3c shows the fabricated flow sensor.

The flow sensor comprised of an 8 mm tall and 400 µm diameter cilia-inspired cylindrical pillar formed from titanium. The high-aspect-ratio (20) titanium pillar, mimicking cilia, enables an enhanced sensitivity in the flow sensor; as compared to the conventional MEMS-based cilia-inspired flow sensors with shorter cilia-like pillar structures, that fall within the flow boundary layer, and hence experience a reduced velocity [5]. The cilia-inspired titanium pillar, which is rooted at the center of the suspended circular CNF membrane (secured using a non-conductive epoxy, EPO-TEK H70E), is free to move at its distal tip. Flows around the sensor would generate a drag force on the titanium pillar owing to fluid–structure interaction, which would lead to a displacement in the suspended CNF membrane on which the pillar is rooted. The displacement of the suspended membrane leads to a change in the electrical conductance path in the CNF percolation network, signifying an overall resistance change which can be measured with an accompanying Wheatstone bridge circuit to which the flow sensor is connected.

#### 2.2.2. Experimental Setup for Steady-State Flow Calibration

For conducting the steady-state airflow calibration tests, a custom in-house built wind tunnel was employed. The wind tunnel was capable of generating a steady-state laminar airflow of up to a velocity of 10 m/s. The lower airflow velocity generation threshold of the wind tunnel was 1.8 m/s, which restricted the accurate determination of the lower detection threshold of the sensor for steady-state flows. The cilia flow sensor was secured in the test section at the center of the wind tunnel, and appropriate provisions were made to acquire the output from the sensor.

#### 2.2.3. Experimental Setup for Oscillatory Flow Calibration

In the past, researchers have extensively studied and employed dipole stimuli for assessing the oscillatory flow sensing performance of cilia-inspired flow sensors [5,7,8]. To assess its dynamic flow sensing performance, the cilia flow sensor was subjected to oscillatory water flow patterns, generated by employing an 8 mm sphere connected to a permanent magnet mini-shaker (acquired from Brüel & Kjær), by a 120 mm long and 2 mm diameter steel rod. The sphere was driven at a 35 Hz frequency, using a function generator connected to a power amplifier (Brüel & Kjær model number 2718). The relationship between the root-mean-square (RMS) velocity of the vibrating sphere and the sinusoidal driving voltage and frequency, has been characterized in the past by employing laser Doppler vibrometry (LDV) [7]. 

#### 2.2.4. Data Acquisition

For all the experiments involving the CNF flexible sensor and cilia flow sensor, the sensors were connected to appropriately designed Wheatstone bridge circuits, powered by a 9 V constant DC power supply. The resistance of the CNF-cilia sensors developed in this work was approximately 330 Ω; and consequently, the fixed resistors of the Wheatstone bridge circuit were chosen as 330 Ω in order to balance the circuit, creating a zero output in the presence of no external flow. A 0−1 kΩ variable resistor was used for balancing the bridge circuit. The unamplified voltage outputs from the Wheatstone bridge circuits were continuously monitored using a National Instruments data acquisition card (NI-DAQ UBS-6009), and logged using the NI Signal Express software at a sampling rate of 2 kHz.

## 3. Results

### 3.1. Characterization of CNF Bundle-Based Flexible Sensors

**Figure 4:**
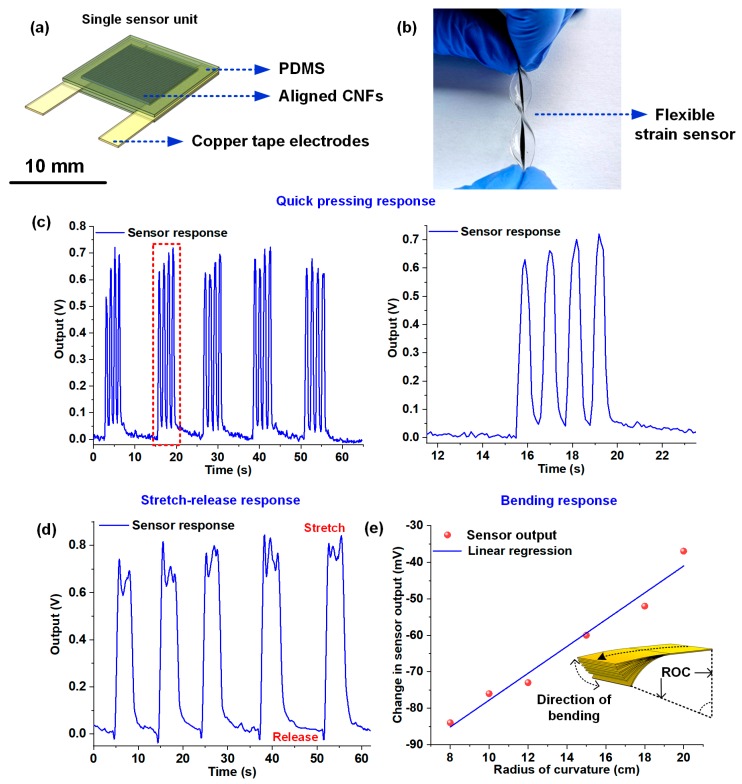
Response plots of the CNF bundle-based flexible sensors: (**a**) Schematic representation of the flexible and stretchable sensor used for the experiments; (**b**) Image of the fabricated CNF-PDMS flexible sensor; (**c**) Plot showing the response of the sensor to four quick press-release cycles followed by a gap of ten seconds. The plot on the right shows the zoomed-in sensor response in the time interval 11–23 seconds; (**d**) Plot showing the response of the sensor to a series of stretch-release cycles; (**e**) Plot showing the change in voltage drop across the sensor at different bending ROCs.

The freestanding CNF bundles were bonded to copper tape electrodes on two edges and encapsulated by PDMS to fabricate flexible and stretchable piezoresistive sensors (Figure 4a,b). To demonstrate the suitability of the sensors for flexible electronic applications, a series of tests comprising of quick tapping, stretching, and controlled bending was conducted. Figure 4b shows the fabricated flexible and stretchable sensor. The sensor was tested for compressive pressing, tensile stretching, and controlled bending to assess its sensing performance for possible applications in wearable and flexible devices. For the first experiment involving press-release cycles, the sensor was pressed four times in quick succession, followed by a gap of ten seconds. The process was repeated five times, and Figure 4c shows the response plot acquired from the sensor while subjecting it to the quick press-release cycles. The zoomed-in plot on the right (Figure 4c) shows the sensor response in the time interval of 11–23 seconds. Overall, a good consistency in the sensor response, with respect to the pressure stimulus, was observed. The sensor was also subjected to a series of stretch-release cycles to demonstrate its capability to detect tensile strains. The experiment involved stretching the flexible sensor and holding it for at least five seconds, followed by release. The sensor response was logged continuously and presented in Figure 4d. To demonstrate the capability of the CNF sensing element in detecting bending, a CNF bundle was sandwiched between two layers of optically clear adhesive (OCA) films, and copper tape electrodes were used to acquire the output. The sensor was subjected to static bending at six different radii of curvatures (ROCs) (8, 10, 12, 15, 18, and 20 cm). The sensor was subjected to a fixed bias current of 1mA with a constant current source, and the voltage across the bundle was recorded using a desktop digital multimeter (HP 34401a). To have calibrated bending response results, 3D printed arc brackets having diameters 8, 10, 12, 15, 18, and 20 cm were used. The sensor was wrapped around each of those brackets, and the voltage changes were measured, as presented in Figure 4e. The unstrained sensor, biased with a 1mA constant current, demonstrated an initial voltage drop of 1.894 volts. Upon bending the sensor at the different radius of curvatures, the conductive path in the bundle comprising of the nanofiber percolation network changed, leading to a reduction in the bundle resistance which manifested as an overall reduction in the voltage drop (with the increase in bending curvature angle) across the bundle, as evident from Figure 4e. The results from the sensing experiments establish the potential of the CNF bundle for the fabrication of flexible and stretchable sensors.

### 3.2. Cilia Flow Sensor Testing and Characterization

**Figure 5:**
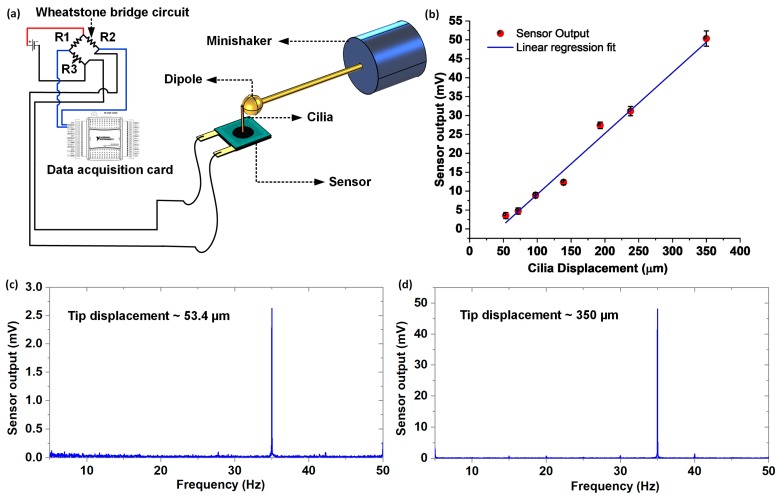
Tip displacement versus sensor response characterization: (**a**) Schematic representation of the experimental setup used for the test, to characterize the tip displacement response of the flow sensor; (**b**) Plot showing sensor output versus the cilium displacement; (**c**,**d**) FFT amplitude plot showing the sensor responses at 35 Hz for two different oscillatory amplitudes (53.4 and 350 µm).

The sensor was characterized for both static flow (in the air) and dynamic flow sensing (in water), to assess its flow sensing performance. The biological cilia observed in nature not only work as flow sensors, but also as touch and vibration sensors. For instance, the cilia found in the cockroaches and crickets developed into drum-like shapes, which are sensitive towards ground vibration [22]. In the past, biological cilia were mimicked to not only work as flow sensors, but also as touch and vibration sensors [23]. To understand the relationship between cilium displacement and sensor output, an oscillatory stimuli response test involving a controlled displacement of the cilium (and simultaneous logging of the voltage response) by employing a vibrating sphere, was conducted. Figure 5a schematically represents the experimental setup employed for the tip displacement response test involving a minishaker connected to a dipole (details of the experimental setup were discussed previously in the materials and methods section). The dipole (at rest) was positioned to touch the tip of the cilium, and was driven (in a direction perpendicular to the axis of the cilium) by the minishaker at a constant frequency of 35 Hz, with seven different amplitudes in the range 50–350 µm. The tests were repeated thrice for each of the individual oscillatory amplitude points. The oscillatory response from the sensor was acquired, and the Fast Fourier Transform (FFT) operation was conducted, where the resulting peak at 35 Hz was used to extract the sensor voltage response for each of the oscillatory amplitude points. The sensor demonstrated a clearly discernable peak at 35 Hz for each of the tested amplitudes. As evident from Figure 5b, the sensor demonstrated a linear response for cilia displacements up to 350 µm, with a lower detection threshold of approximately 50 µm. Figure 5c,d show the FFT amplitude plots for the sensor driven with a constant frequency of 35 Hz, at two different amplitudes of 53.4 µm and 350 µm respectively.

**Figure 6:**
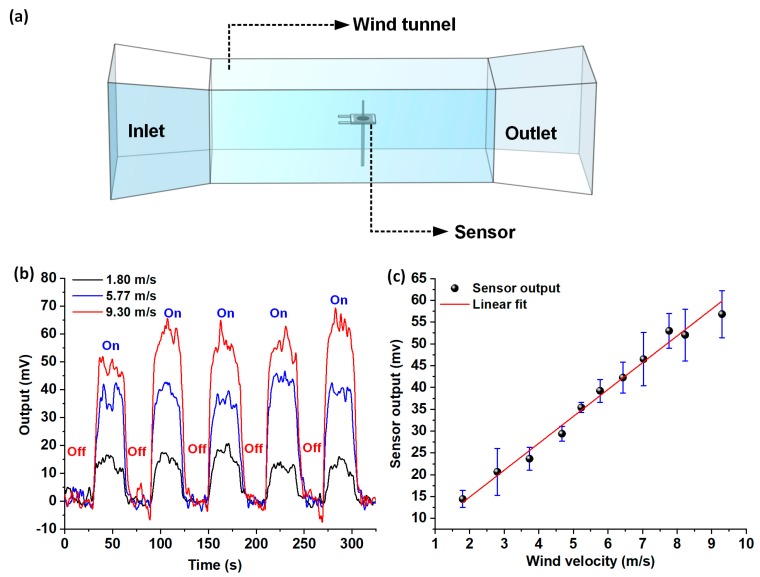
Steady-state airflow sensing characterization: (**a**) Schematic representation of the experimental setup involving the wind tunnel for steady-state flow calibration; (**b**) Superimposition plot showing the voltage response of the sensor for three different airflow velocities; (**c**) Plot showing the Wheatstone bridge output versus the airflow velocity, in the range 1.8–9.3 m/s.

The flow sensor was characterized for steady-state wind flow sensing in the velocity range of 1.8–9.3 m/s. Figure 6a shows the schematic representation of the custom in-house built wind tunnel setup, employed for conducting the steady-state flow calibration experiments. At each of the individual velocity points, the wind tunnel was turned on and off five times in a periodic fashion, with each cycle having a duration of 60 seconds; the experiment was repeated thrice. The sensor response from the Wheatstone bridge circuit was recorded. The Wheatstone bridge outputs for individual flow velocities were acquired, and average peak heights of the steady-state response signals were determined using OriginPro data analysis software. The peak heights were averaged over the five on-off cycles (per experimental run), and standard deviations were calculated for all the individual velocity points over the three runs of the experiment. Figure 6b shows the superimposed plots of the raw Wheatstone bridge outputs acquired for three different velocities (1.8, 5.77, and 9.3 m/s). Figure 6c shows the voltage response of the sensor to steady-state flow velocities in the range of 1.8–9.3 m/s, with the error bars representing standard deviation. The steady-state response of the sensor was found to be linear, with a high sensitivity of 6.16 mV/(m s^−1^).

**Figure 7:**
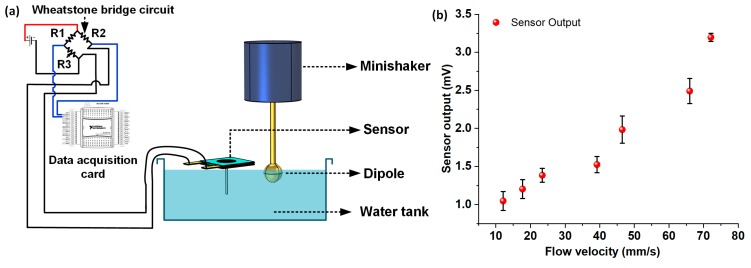
Oscillatory flow sensing characterization underwater: (**a**) Schematic representation of the experimental setup involving the dipole setup for oscillatory flow calibration; (**b**) Plot showing sensor output versus the oscillatory flow stimuli.

Finally, the flow sensor was assessed for oscillatory flow sensing performance, by submerging the cilium in deionized (DI) water and generating oscillatory flow stimuli by employing the dipole-minishaker setup described previously. Figure 7a shows the schematic representation of the experimental setup employed for the oscillatory flow calibration experiments. The dipole was entirely submerged below the surface of the water and driven at a constant frequency of 35 Hz to generate oscillatory dipole stimuli. The sensor was placed in such a way that only the cilium of the sensor was submerged in water to avoid contact of the suspended CNF diaphragm with DI water. The tip of the cilium was kept at a distance of 1 cm from the edge of the dipole to avoid any physical contact between the vibrating sphere and the cilium tip during the flow calibration experiments. The root-mean-square (RMS) velocity of the flow was swept in the range of 12–72 mm/s by tuning the RMS driving voltage input to the minishaker, and the sensor response from the accompanying Wheatstone bridge circuit was recorded. The data were treated with bandpass filters to get rid of the power supply interference, and FFT operations were carried out on the sensor response data to determine the peak sensor response amplitude at each of the individual oscillatory flow velocity points. For oscillatory flow velocities below 12 mm/s, no clearly discernable peaks (from the device noise floor) were observed at 35 Hz. The experiments were repeated thrice for each of the velocity points, and the standard deviations were calculated. Figure 7b shows the calibration response plot of the sensor for oscillatory flow, with error bars representing standard deviation. As observed from the oscillatory flow response calibration curve, the sensor demonstrated a linear response in the flow velocity range of 12–66 mm/s. A linear regression fit was conducted in the linear velocity range (12–66 mm/s) to determine the oscillatory flow sensitivity of the sensor. The sensor demonstrated a sensitivity of 26 mV/(m s^−1^) in the linear regime of the flow calibration plot, with a lower detection threshold limit of 12.1 mm/s.

## 4. Conclusions and Future Work

In conclusion, we presented a simple process flow for fabricating inexpensive, yet sensitive and flexible, artificial cilia flow sensors. The proposed sensor comprised of a cilia-inspired high-aspect-ratio titanium pillar placed on a suspended CNF membrane sensing element. The CNF bundles were embedded in PDMS to develop stretchable and flexible sensors, to demonstrate the feasibility of their usage in wearable device applications. Tip displacement response tests conducted on the flow sensor revealed a linear voltage response, with a low detection threshold of 50 µm. Flow calibration experiments conducted on the sensor revealed sensitivity figures of 6.16 mV/(m s^−1^) and 26 mV/(m s^−1^) for steady-state airflow and oscillatory waterflow, respectively. Furthermore, the sensor demonstrated a detection threshold of 12.1 mm/s for oscillatory flow tests in water. The simple workflow demonstrated in this work will enable the design and fast development of a future class of inexpensive, yet sensitive, artificial cilia flow sensors, for various applications requiring precise flow measurement and control. 

For precise flow measurement applications, the sensitivity and the lower detection threshold of the sensor are crucial; and it is possible to improve these two parameters further by incorporating drop-casted hydrogel on top of the titanium pillar, to mimic the biological cupula. The biological cupula found in neuromasts of fish increases the amount of drag force exerted on the sensory hair cells, subsequently increasing their sensitivity. Additionally, in the past, we have proposed the incorporation of an array of cilia-inspired sensors into canal neuromast (CN)-inspired biomimetic polymer canals to reduce noise due to environmental factors [24]. Incorporating the cilia flow sensors into appropriately designed biomimetic canals with pores that allow external flows to enter the canal could potentially filter noisy flows. In addition, the canal packages also protect the fragile cilia from mechanical and accidental damage. Tests from our previous work validated the biomechanical high-pass filtering capability of biological CN-inspired canals, subsequently improving the signal to noise ratio and signal selectivity. Hence, an appropriately designed sensor array, with hydrogel cupula and biological CN-inspired packaging, might potentially enhance the signal to noise ratio, sensitivity, resolution, and lower detection threshold of the sensor design proposed in this paper.
